# Limb-girdle muscular dystrophy with severe heart failure overlapping with lipodystrophy in a patient with *LMNA* mutation p.Ser334del

**DOI:** 10.1007/s13353-016-0365-2

**Published:** 2016-09-01

**Authors:** Agnieszka Madej-Pilarczyk, Adam Niezgoda, Magdalena Janus, Romuald Wojnicz, Michał Marchel, Anna Fidziańska, Stefan Grajek, Irena Hausmanowa-Petrusewicz

**Affiliations:** 1 Neuromuscular Unit, Mossakowski Medical Research Centre, Polish Academy of Sciences, Pawinskiego St. 5, 02-106 Warsaw, Poland; 2 Department of Neurology, Poznan University of Medical Sciences, Poznan, Poland; 3 1st Department of Cardiology, Poznan University of Medical Sciences, Poznan, Poland; 4 Department of Histology, School of Medicine with the Division of Dentistry, Medical University of Silesia in Katowice, Zabrze, Poland; 5 1st Department of Cardiology, Medical University of Warsaw, Banacha 1a, Warsaw, Poland

**Keywords:** *LMNA* gene, Lamin A/C, Laminopathy, Limb-girdle muscular dystrophy, Cardiomyopathy

## Abstract

Laminopathies, a group of heterogeneous disorders associated with lamin A/C gene (*LMNA*) mutations, encompass a wide spectrum of clinical phenotypes, which may present as separate disease or as overlapping syndromes. We describe a 35-year-old female in whom a novel sporadic heterozygous mutation c.1001_1003delGCC (p.Ser334del) of the *LMNA* gene was found. The patient presented with overlapping syndrome of heart failure secondary to dilated cardiomyopathy, limb-girdle dystrophy and partial lipodystrophy. Endomyocardial biopsy revealed strong up-regulation of HLA classes I and II antigens on microvessels and induction of the class I antigens on cardiomyocytes. On muscle biopsy, a wide range of fiber sizes and small clusters of inflammatory infiltrations were found. In the rapid progression of heart failure with arrhythmias or conduction defect, accompanied with muscle atrophy and lipodystrophy, the genetic disease should be taken into consideration. In addition, undefined inflammatory response and fibrosis in the heart or skeletal muscle might further justify screening of the lamin A/C gene.

## Introduction

Laminopathies, a group of inherited human diseases that affect mainly tissues of mesenchymal origin, are associated with structural/functional defect of genes that encode the nuclear envelope proteins. The main component of nuclear lamina, located under the inner nuclear membrane, is lamin A/C, encoded by the *LMNA* gene. Mutations in the *LMNA* gene cause a number of clinical phenotypes, including muscular, peripheral neurogenic, lipodystrophies and premature ageing syndromes (Worman and Bonne 2007). Laminopathies may present as separate disease or as overlapping syndromes; however, clear phenotype–genotype correlations have been difficult to establish. The meta-analysis of Carboni et al. (2013) shows that heterozygous missense mutations spreading throughout the *LMNA* gene are responsible for the majority of overlapping laminopathies. Mutated lamin A/C acts in a dominant-negative way on normal protein translated on the healthy allele, altering its stability and interaction with partner proteins. Here, we present a patient with complex clinical syndrome characterized by dilated cardiomyopathy, limb-girdle dystrophy and partial lipodystrophy with an inflammatory response in the muscle and endomyocardial biopsy specimens.

## Case study

**Fig. 1:**
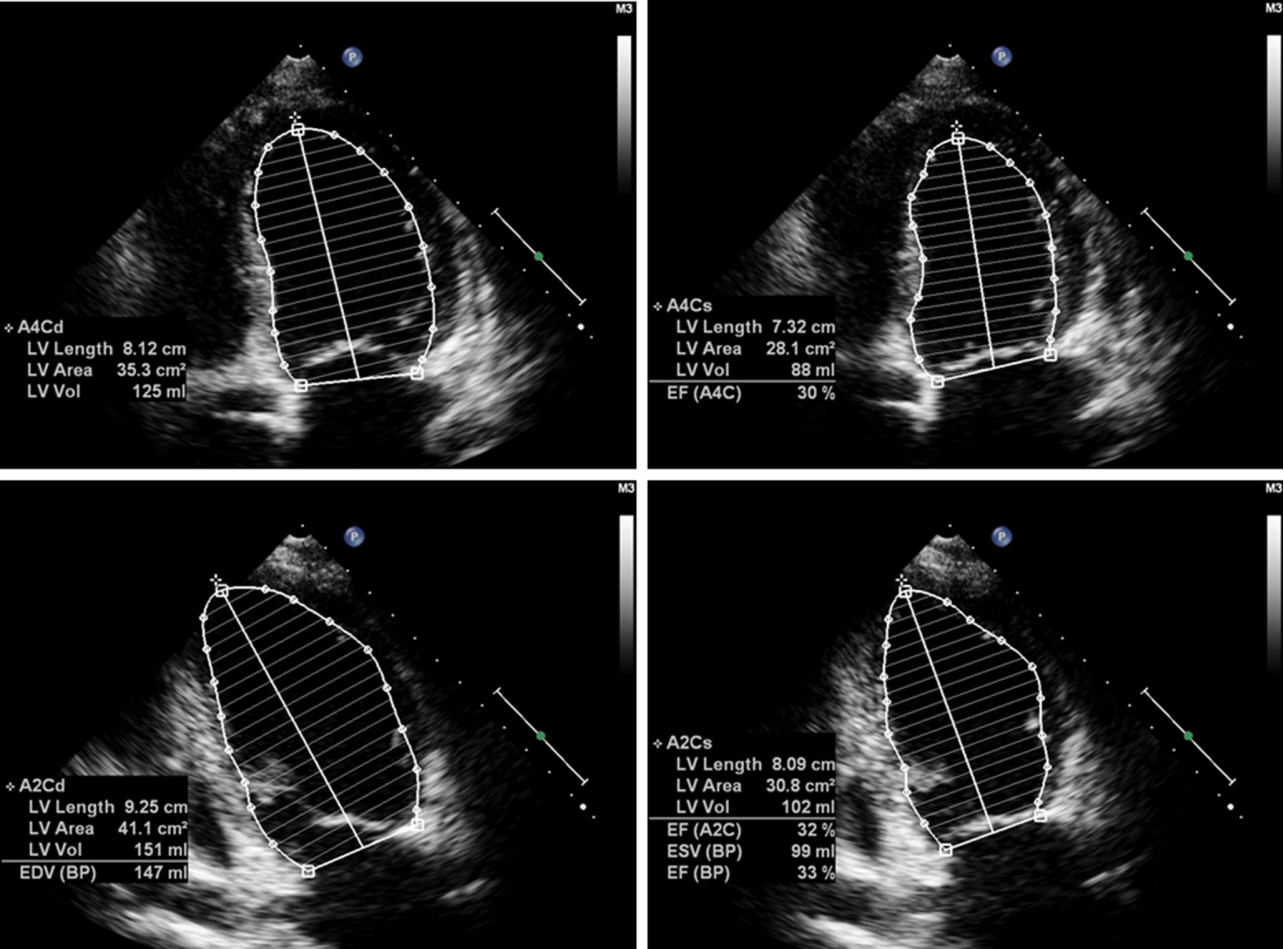
Restrictive cardiomyopathy on echocardiogram: note significantly decreased ejection fraction (EF) of 33 %, increased end-systolic volume (ESV) of 99 ml (normal <50 ml) and end-diastolic volume (EDV) of 147 ml (normal <120 ml)

**Fig. 2:**
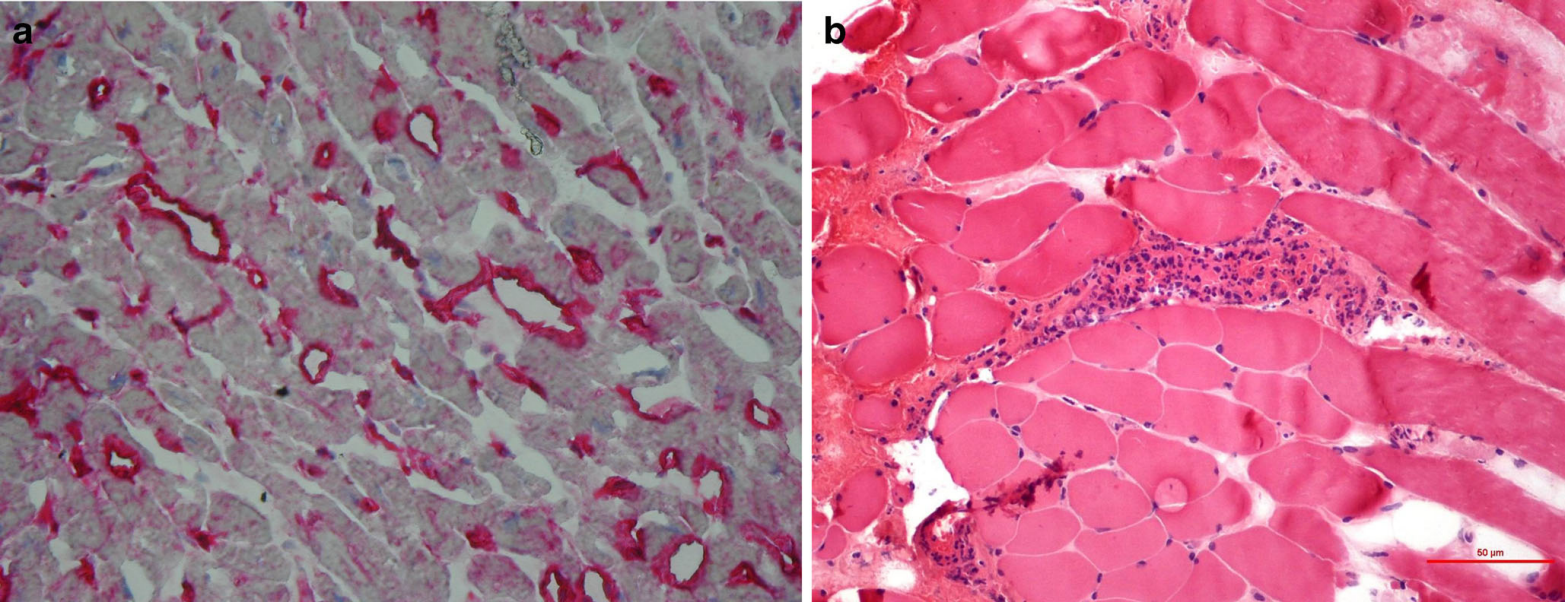
**a** Strong expression (IR = 2+) of HLA-ABC antigens by microvascular endothelium denoted by the red staining (original magnification × 400×). **b** Histopathological evaluation of left m. pectoralis major: focally distributed inflammatory infiltrations, localized mainly in the perivascular area (original magnification × 400×)

A 35-year-old Caucasian female with overlapping laminopathy was found to carry a novel sporadic heterozygous mutation c.1001_1003delGCC (p.Ser334del) of the *LMNA* gene. Although systolic dysfunction was dominated in the clinical presentation, some additional findings helped to establish the final diagnosis of laminopathy. First clinical symptoms such as dyspnea, peripheral edema, palpitations and general weakness occurred at the age of 32 years, suggesting myocardial disease. Electrocardiography revealed atrial fibrillation/flutter and supraventricular tachycardia (SVT) with artio-ventricular block (AVB) 2:1. Echocardiography examination revealed significant systolic dysfunction with left ventricular ejection fraction (EF) of 30 % and pericardial effusion (Fig. 1). The initially considered diagnosis was post-inflammatory dilated cardiomyopathy. For this reason, right ventricular endomyocardial biopsy was performed. Histochemistry excluded acute myocarditis based on the Dallas criteria but showed moderate interstitial fibrosis in the biopsy specimens (Masson’s trichrome stain). Immunohistochemistry revealed strong endothelial expression of HLA classes I and II antigens with concomitant de novo induction of class I on cardiomyocytes (Fig. 2a). At the cardiology clinic, the patient had moderate symptoms of heart failure [New York Heart Association (NYHA) classes II and III], which was stable under oral treatment. The serum markers of myocardial necrosis were negative. The treatment included electric cardioversion (100 J), which allowed temporary restoration of sinus rhythm with AVB 2:1, followed by SVT with AVB 2:1 and 3:1. During the subsequent 2 years, recurrent atrial fibrillation/flutter were observed, which proved to be resistant to electric cardioversion (200 J). In addition, ventricular arrhythmia (pairs, triplets) was registered.

**Fig. 3:**
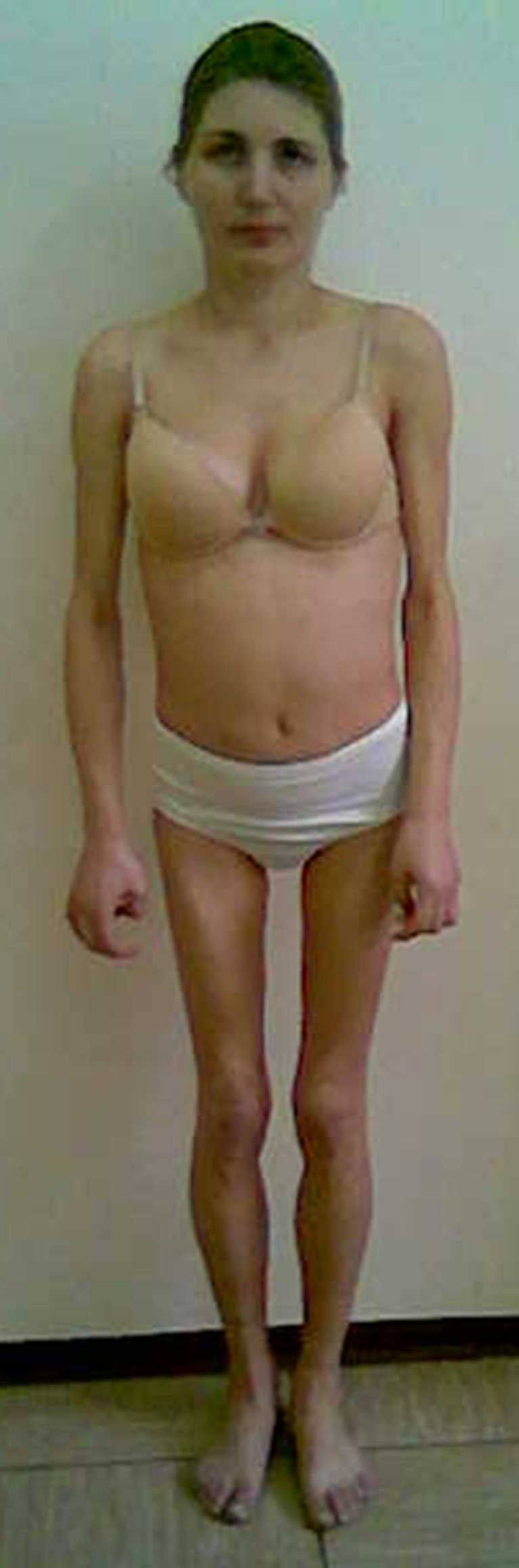
A 38-year-old female: note atrophy of proximal muscles, slight elbow contractures and hyperlordosis and loss of subcutaneous fat on arms and legs

Follow-up physical examinations revealed slowly progressing weakness and atrophy of limb-girdle muscles, noted at first during adolescence, absence of knee reflexes, rigid spine with mild hyperlordosis and slight elbow and knee contractures. Electromyography done twice at the ages of 20 years and 34 years did not show myopathic pattern; however, histopathological examination of muscle (m. pectoralis major) revealed variability of fiber diameter and focally distributed inflammatory infiltrations (Fig. 2b). Medical history review of infertility, amenorrhea and excision of bilateral endometrial ovarian cysts at age 32 years, as well as prominent loss of subcutaneous fat on arms and legs, might be consistent with partial lipodystrophy features (Fig. 3). Glucose and lipids were within the normal ranges. All those findings made a potential background of overlapping laminopathy very probable.

**Fig. 4:**
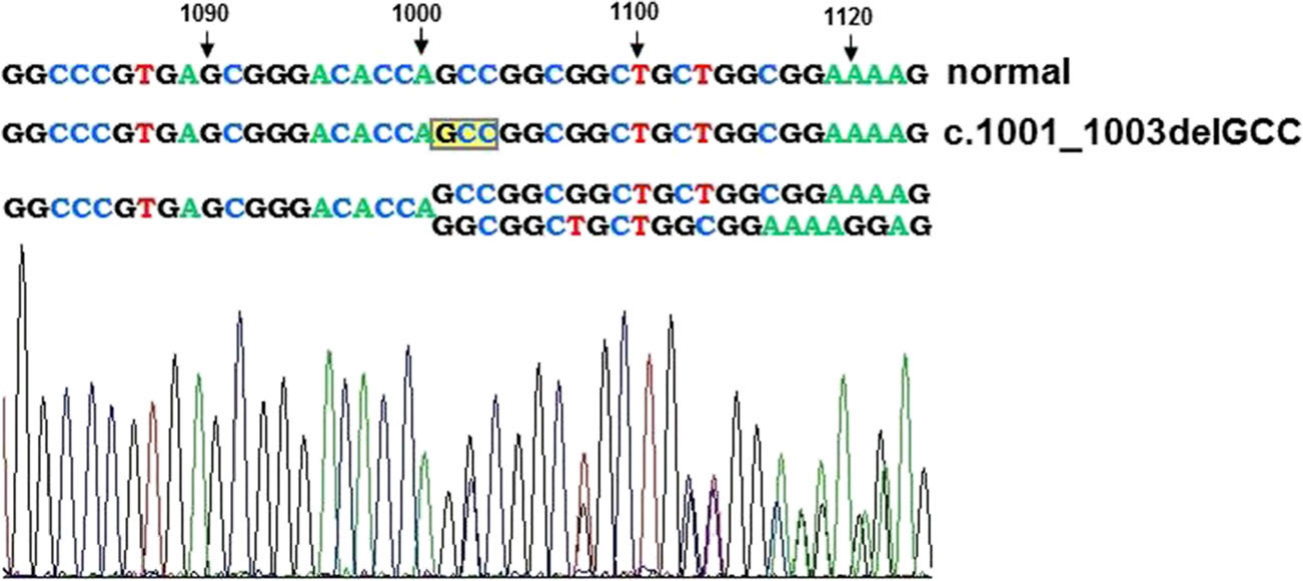
Direct sequencing showing heterozygous in-frame deletion c.1001_1003delGCC (p.Ser334del) in exon 6 of the *LMNA* gene

Once written informed consent was received, we performed genetic analysis. The study was approved by the local Bioethical Committee. Genomic DNA was extracted from peripheral blood lymphocytes using the salting-out procedure. All 12 exons of *LMNA* and exon–intron junctions were amplified by polymerase chain reaction (PCR), sequenced using the BigDye Terminator Sequencing Ready Reaction Kit (Applied Biosystems) and analysed on an ABI PRISM 373 fluorescent DNA sequencer (Applied Biosystems). Primer sequences and PCR protocols are available upon request. In fact, direct DNA sequencing of 12 exons and exon–intron boundaries of the *LMNA* gene revealed a heterozygous in-frame deletion c.1001_1003delGCC (p.Ser334del) of the *LMNA* gene (Fig. 4).

A final diagnosis of laminopathy guided follow-up cardiological therapy. Because of the high risk of sudden cardiac death due to significant systolic dysfunction and mutation in the *LMNA* gene, a cardioverter-defibrillator (Biotronik Setrox S 53) was implanted. However, despite optimal therapeutic strategy, progression of heart failure had been observed. At the age of 35 years, the patient underwent heart transplantation and, 5 years after surgery, its effect remained satisfactory. Skeletal muscle disease is gradually progressing, leading to marked muscle atrophy with concomitant quite well preserved strength. In addition to fat loss from the extremities, accumulation of subcutaneous fat on the neck, face and trunk, typical for partial lipodystrophy, is seen. Regular menses returned after transplantation, so it seems that amenorrhea might be partially associated with poor nutrition status in the period of heart failure.

## Discussion

There are several reports of laminopathies characterized by complex phenotype, composed of lipodystrophy, cardiac disease and muscular dystrophy. Two of three patients described in the paper of van der Kooi et al. (2002), bearing missense mutations in exon 9 of the *LMNA* gene, had features of partial lipodystrophy and muscle weakness, spine rigidity and joint contractures observed since adolescence. In contrast to the patient described here, their cardiac presentation included atrial fibrillation without symptoms of cardiomyopathy. The third patient, having mutation in exon 1 of the *LMNA* gene, had partial lipodystrophy and congestive heart failure with conduction defect, but the first signs of cardiac disease occurred later than in our patient, i.e. in the fifth decade of life. In contrast to our patient, partial lipodystrophy was accompanied by hypertriglyceridemia and diabetes mellitus, and skeletal muscles were not affected. Two other families with familial partial lipodystrophy (FPLD) and cardiomyopathy with conduction system defects and proximal muscle weakness, caused by missense mutations in exon 1 of the *LMNA* gene, were described by Garg et al. (2002), although cardiac manifestation was not predominant in these cases.

The *LMNA* variant c.1001_1003delGCC (p.Ser334del) in the patient presented here was reported in the meta-analysis of Carboni et al. (2013), although without a detailed description of its clinical consequences. It is localized in exon 6 of the *LMNA* gene and it results in the deletion of serine in position 334 of the lamin A/C. This residue is located in the central alpha-helical rod domain of the protein and it is responsible for lamin A homodimer formation. As interhelical salt bridges are between residues p.Glu330 and p.Arg331 and between p.Lys331 and p.Glu342, we might presume that the deletion of Ser334 impaired stereochemical interaction of the protein (Strelkov et al. 2004). This pathomechanism of laminopathy was postulated by other authors in patients with missense or frameshift mutations in the 2B coil domain of lamin A/C (Benedetti et al. 2007; Volpi et al. 2010). Deletions in the *LMNA* gene are rare and responsible for only 6 % of known mutations of this gene. There was a report of missense point mutations in position 1003, i.e. c.1003C>T (p.Arg335Trp), which was associated with complex phenotype, including muscle weakness, atrial fibrillation, hypertriglyceridemia and acro-osteolysis, presented by Drouin et al. during the 54th Annual Meeting of the American Society of Human Genetics (ASHG) in 2004. Other reported mutations located in close vicinity to nucleotide position 1000 of the *LMNA* gene have been shown to result in cardiomyopathy with conduction disturbances and, finally, sudden cardiac death, despite the implantation of a cardioverter-defibrillator (c.992G>A, p.Arg331Gln) (Møller et al. 2009) and in Emery–Dreifuss muscular dystrophy (EDMD) with early cardiac onset (c.1007G>A, p.Arg336Gln) (Raffaele Di Barletta et al. 2000; Sanna et al. 2003). Complex phenotype comprised of limb-girdle muscular dystrophy (LGMD) with atrial fibrillation and tachy-brady syndrome, features of FPLD, short stature and facial dysmorphy was described by Mercuri in a patient with mutation p.E358K in exon 6 of the *LMNA* gene. The same mutation was responsible for the EDMD phenotype in four other patients. Interestingly, in one of them, mild up-regulation of HLA class I on the sarcolemma of mature fibers was found in muscle biopsy specimen, which might be suggestive for inflammatory myopathy. In our patient, similar up-regulation and cardiomyocyte induction of HLA class I antigens were observed. In addition to the variability of fiber diameter in the skeletal muscle biopsy consistent with dystrophic changes, focally distributed inflammatory infiltrations, localized mainly in the perivascular area, were seen. A similar observation has already been made by Komaki et al. (2011), who described perivascular cuffing and/or endomysial/perimysial lymphocyte infiltration in patients with *LMNA* mutations, initially presenting signs of infantile onset inflammatory myopathy and then developed joint contractures and/or cardiac involvement. Hence, it is suggested that inflammation might be an additional pathological process in affected cardiac and skeletal muscles in laminopathy. Significant interstitial myocardial fibrosis, which was seen in our patient in the period of fast progressing heart failure, is consistent with observations of other authors in patients with cardiomyopathy associated with defects of the *LMNA* gene (Fatkin et al. 1999; van Tintelen et al. 2007; Holmström et al. 2011). Replacement of cardiomyocytes by connective tissue, especially of the basal septum, leads to conduction abnormalities and atrial and ventricular arrhythmia, which is usually the first manifestation of cardiac disease in laminopathy.

In conclusion, upon the rapid progression of heart failure with arrhythmias or conduction defects, accompanied by muscle atrophy and lipodystrophy, the genetic disease should be taken into consideration, even in negative familiar history. Myocardial fibrosis or unspecific inflammatory signs in microscopic examination of heart or skeletal muscle might further justify screening of the *LMNA* gene.
